# Supplementary material for: Barium content of Archaean continental crust reveals the onset of subduction was not global
Source: Nat Commun. 2022 Nov 2;13:6553. doi: 10.1038/s41467-022-34343-0 (PMC9630499; doi:10.1038/s41467-022-34343-0)
Supplement: Supplementary file 1 — Supplementary Information [file 41467_2022_34343_MOESM1_ESM.pdf]

Barium content of Archaean continental crust reveals the onset of subduction was not global

Guangyu Huang*, Ross N. Mitchell*, Richard M. Palin, Christopher J. Spencer, Jinghui Guo

*Corresponding authors: huangguangyu@mail.iggcas.ac.cn; ross.mitchell@mail.iggcas.ac.cn

Supplementary information

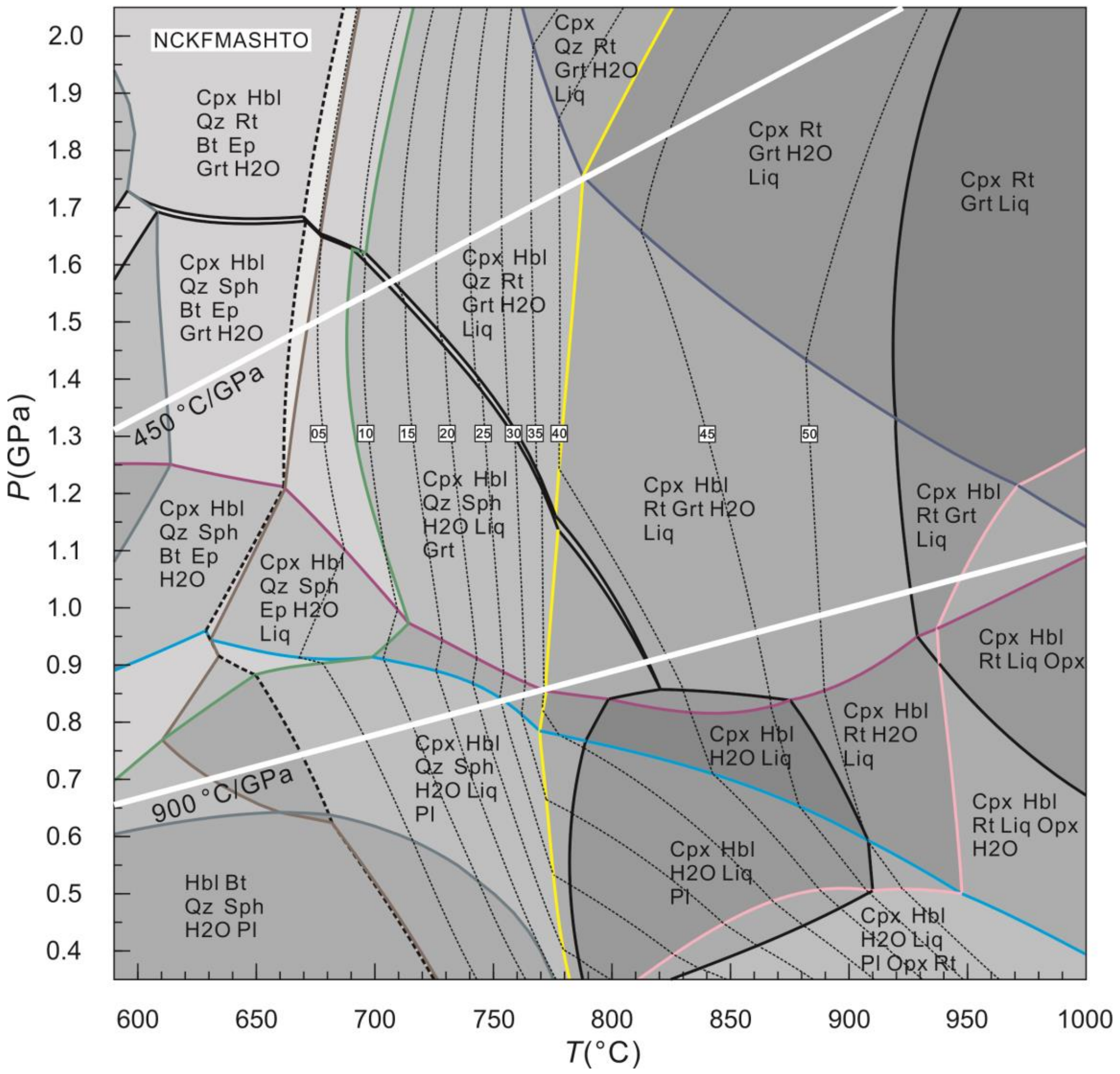

Supplementary Figure 1. Full phase diagram for averaged Archaean basaltic composition, with water content ensuring fluid-present melting. Cpx, clinopyroxene; Hbl, hornblende; Qz, quartz; Rt, rutile; Bt, biotite; Ep, epidote; Grt, garnet; H2O, aqueous fluids; Sph, sphene; Pl, plagioclase; Liq, melt; Opx, orthopyroxene.

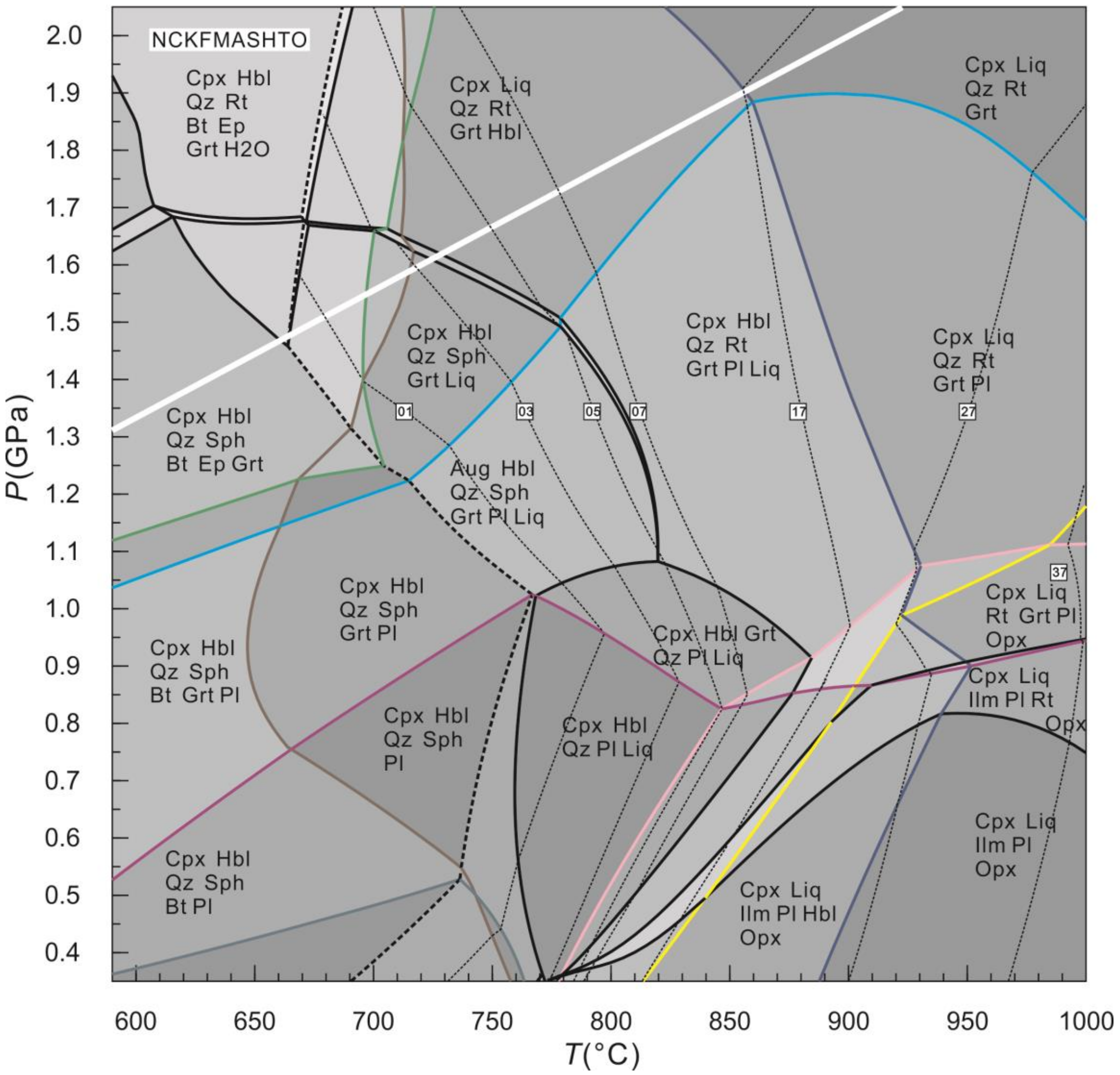

Supplementary Figure 2. Full phase diagram for averaged Archaean basaltic composition, with water content allowing just saturated at the solidus along 450 °C GPa^{-1}(Huang, G., Palin, R., Wang, D. et al. Open-system fractional melting of Archean basalts: implications for tonalite–trondhjemite–granodiorite (TTG) magma genesis. Contrib Mineral Petrol 175, 102 (2020). https://doi.org/10.1007/s00410-020-01742-9). Cpx, clinopyroxene; Hbl, hornblende; Qz, quartz; Rt, rutile; Bt, biotite; Ep, epidote; Grt, garnet; H2O, aqueous fluids; Sph, sphene; Pl, plagioclase; Liq, melt; Opx, orthopyroxene; Ilm, ilmenite.

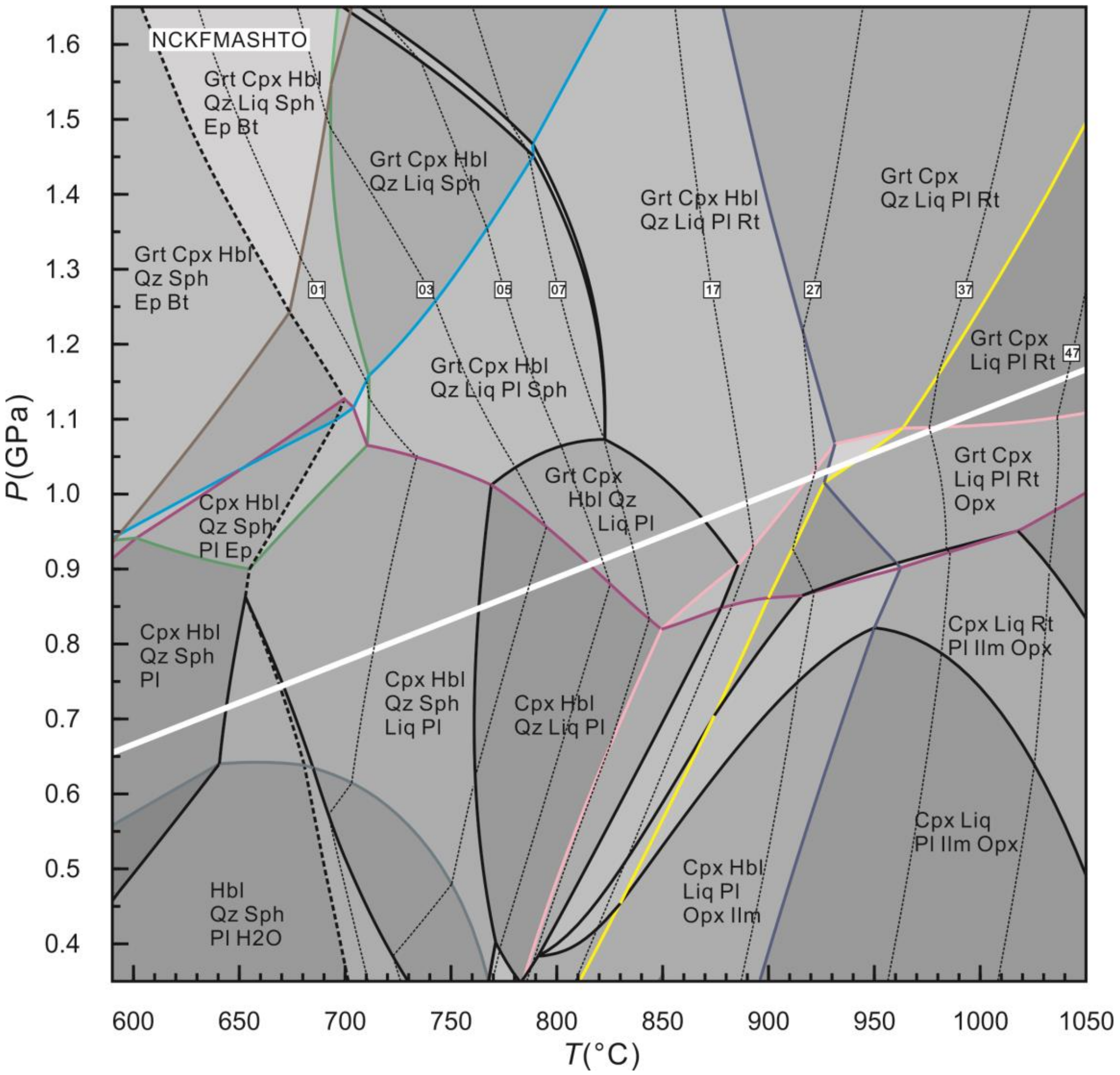

Supplementary Figure 3. Full phase diagram for averaged Archaean basaltic composition, with water content allowing just saturated at the solidus along 900 °C GPa^{-1}(Huang, G., Palin, R., Wang, D. et al. Open-system fractional melting of Archean basalts: implications for tonalite–trondhjemite–granodiorite (TTG) magma genesis. Contrib Mineral Petrol 175, 102 (2020). https://doi.org/10.1007/s00410-020-01742-9). Cpx, clinopyroxene; Hbl, hornblende; Qz, quartz; Rt, rutile; Bt, biotite; Ep, epidote; Grt, garnet; H2O, aqueous fluids; Sph, sphene; Pl, plagioclase; Liq, melt; Opx, orthopyroxene; Ilm, ilmenite.

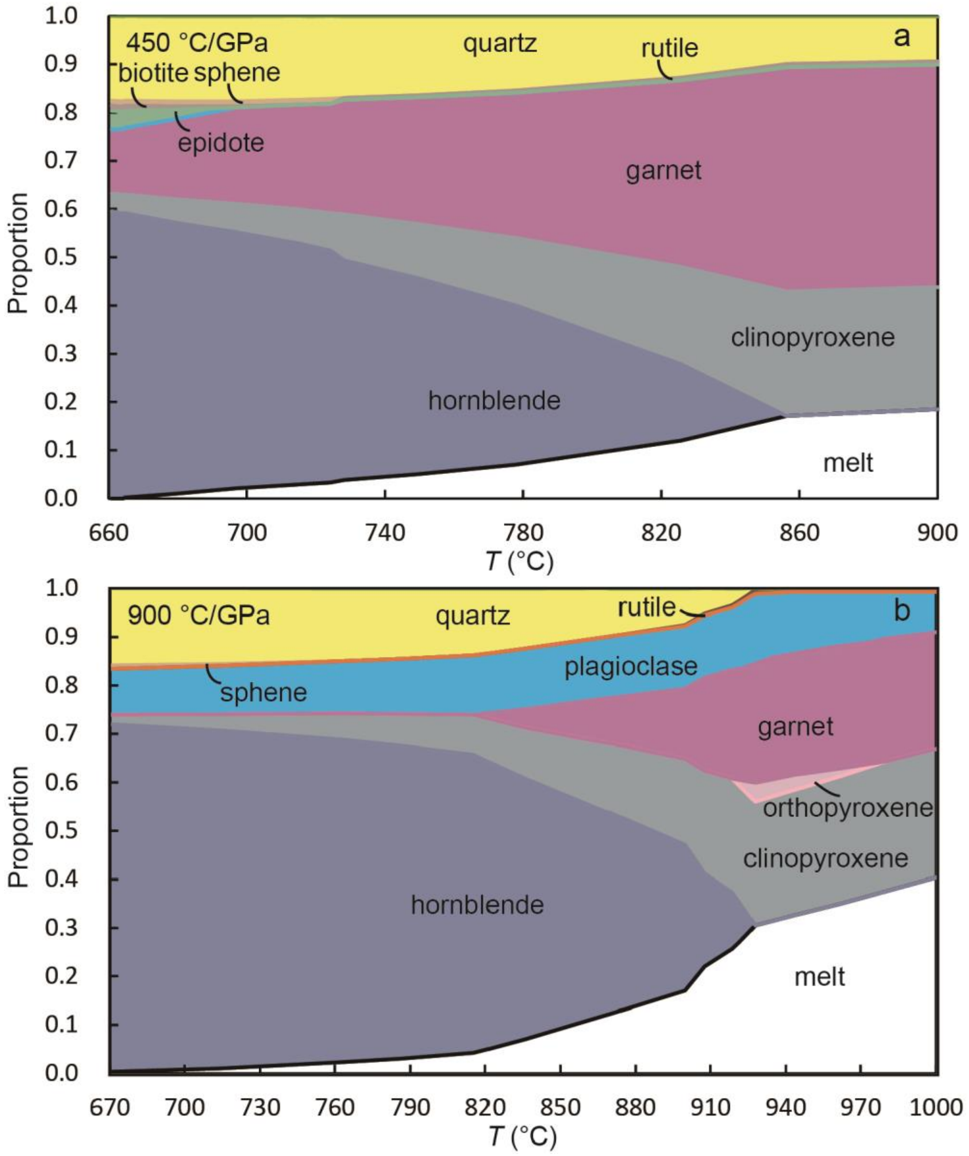

Supplementary Figure 4. Modeled mineral and melt proportions along (a) 450 °C GPa^{-1} and (b) 900 °C GPa^{-1} during fluid-absent melting(Huang, G., Palin, R., Wang, D. et al. Open-system fractional melting of Archean basalts: implications for tonalite–trondhjemite–granodiorite (TTG) magma genesis. Contrib Mineral Petrol 175, 102 (2020). https://doi.org/10.1007/s00410-020-01742-9).

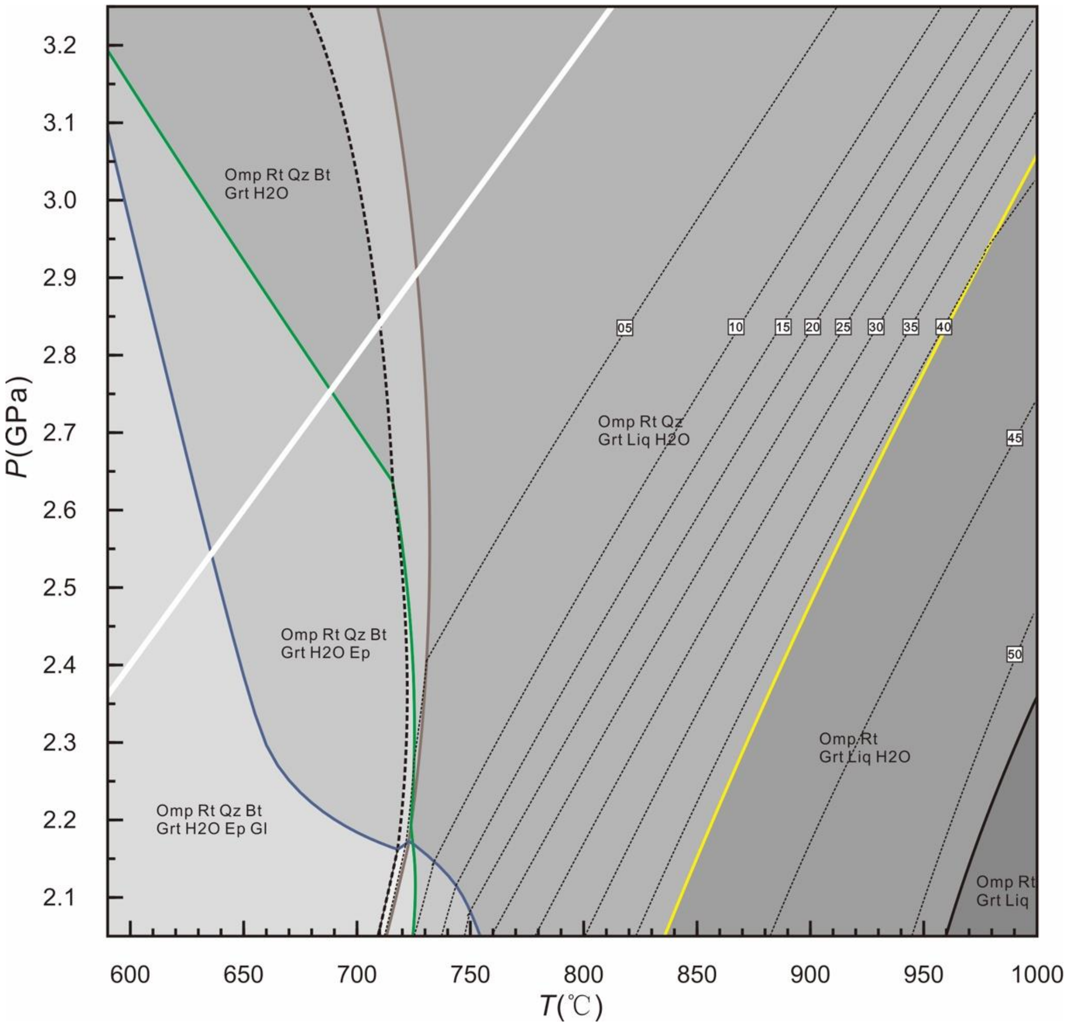

Supplementary Figure 5. Full phase diagram for averaged Archaean basaltic composition, with water content ensuring fluid-present melting along 250 °C GPa^{-1}. Omp, omphacite; Rt, rutile; Qz, quartz; Bt, biotite; Grt, garnet; H2O, aqueous fluids; Ep, epidote; Gl, glaucophane; Liq, melt.

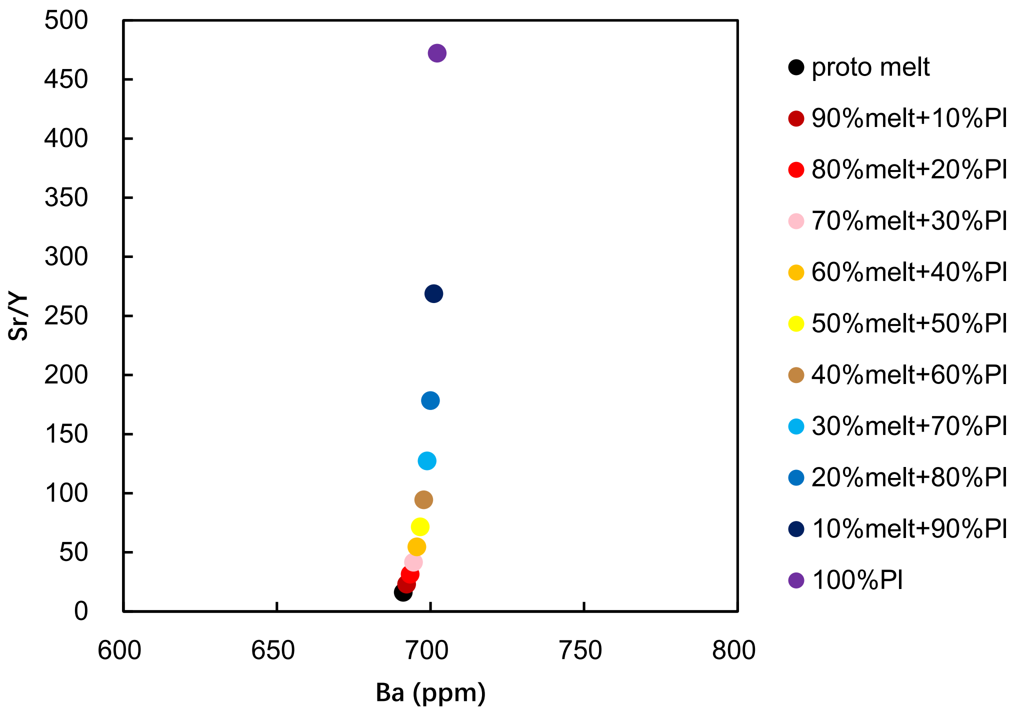

Supplementary Figure 6. Calculated results of different degrees of plagioclase accumulation. The proto melt composition comes from 7 mol.%. fluid-present melting at 900 °C GPa^{-1} in this study. The plagioclase composition is calculated in equilibrium with the melt, based on the partition coefficient list in Supplementary Table 1. Pl, plagioclase.

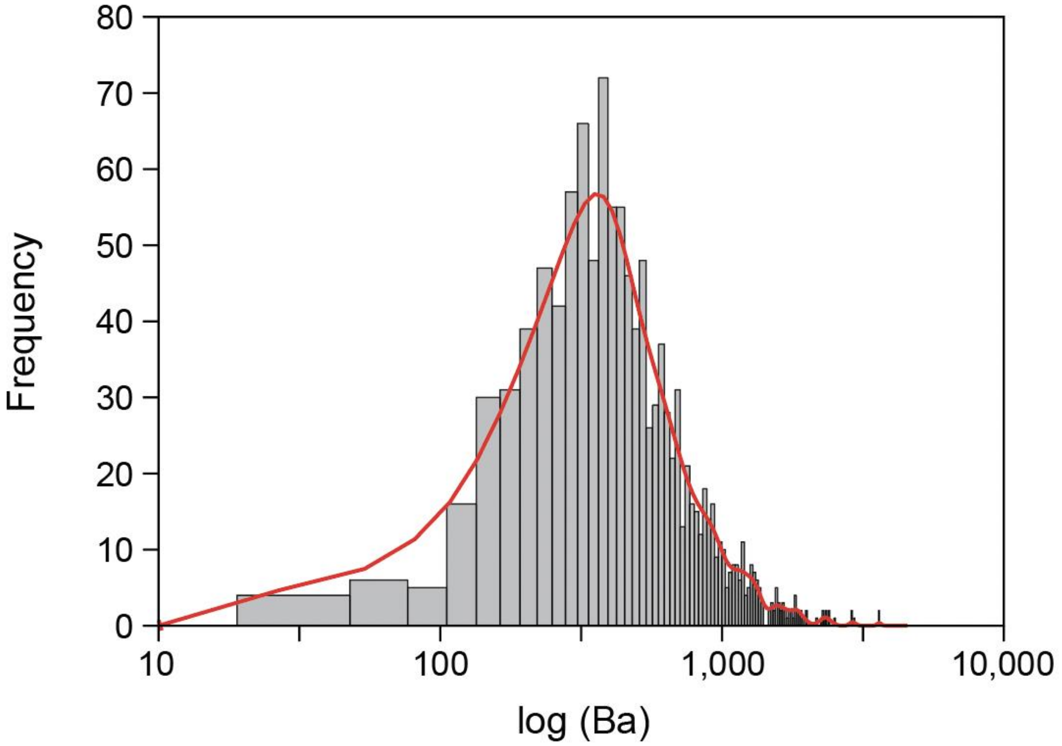

Supplementary Figure 7. Histogram of global Ba data in Archaean TTG. Note log-scale on the *x*-axis. Red line is the kernel density estimation (KDE). The Ba data are an example of a log-normal distribution (i.e., normally distributed in log space) and are therefore converted to log(Ba) for statistical analysis through time. Two outliers with low Ba values <<10 were excluded from this plot.

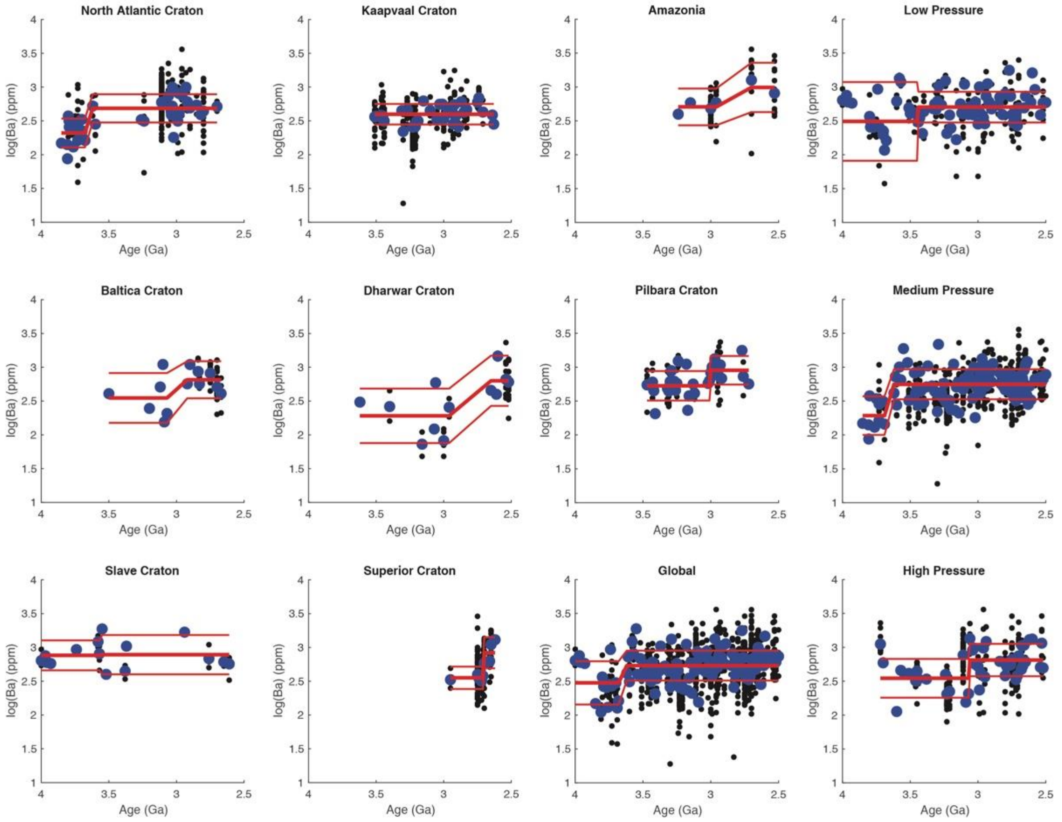

Supplementary Figure 8. Ba contents of TTGs of specific cratons though the Archaean. Black points are individual data and blue points are averages for rocks of identical age. We excluded single datums (blue "averages" with only one black data point) that are outliers either in age and/or Ba value.

Supplementary Table 1. The partition coefficients used for trace element modelling

	Hbl	Cpx	Opx	Pl	Bt	Rt	Sph	Ep	Zrn	Apa
La	0.319	0.028	0.0003	0.358	0.02	0.0057	4.73	2.05	26.6	12
Yb	1.79	0.635	0.125	0.094	0.11	0.0126	3.02	2.96	490	13
Sr	0.389	0.032	0.047	4	0.1	0.036	2.68	2	20	1.4
Y	2.47	0.603	0.054	0.138	0.07	0.0118	5.42	4.3	80	17.5
Nb	0.8	0.007	0.01	0.239	0.085	158	2.2	0.226	50	0.05
Ta	0.38	0.028	0.126	0.053	0.107	173	6.55	0.226	50	0.05
Cr	4.7	0.582	7.97	0.15	6.8	0	0	0.0029	0	0.2
Ni	6.12	5.96	7.35	1.73	1.75	0	0	0.1	0	0.4
Ba	0.046	0.006	0.047	1.016	6	0.0043	1.5	0.408	4	0.45

Notes: D values follow ref. [1].

Supplementary Table 2. Intitial bulk compositions used for phase equilibria modelling (wt.%)

	Fluid present	Fluid absent (450 °C/GPa)	Fluid absent (900 °C/GPa)
	Fig. 2, S1, S5	Fig.S2, S4a	Fig. S3, S4b
H2O	7.04	1.45	1.33
SiO_2	49.4	52.4	52.4
Al_2O_3	13.9	14.7	14.7
CaO	9.57	10.1	10.2
MgO	7.13	7.56	7.56
FeO	8.86	9.40	9.41
Fe_2O_3	1.08	1.15	1.15
K_2O	0.253	0.268	0.269
Na_2O	1.97	2.09	2.09
TiO_2	0.818	0.868	0.869

Supplementary Reference

1. Bédard, J. H. A catalytic delamination-driven model for coupled genesis of Archaean crust and sub-continental lithospheric mantle. *Geochim. Cosmochim Acta* **70**, 1188–1214 (2006).
