# Supplementary material for: Barium content of Archaean continental crust reveals the onset of subduction was not global
Source: Nat Commun. 2022 Nov 2;13:6553. doi: 10.1038/s41467-022-34343-0 (PMC9630499; doi:10.1038/s41467-022-34343-0)
Supplement: Supplementary file 3 — Description of Additional Supplementary Files [file 41467_2022_34343_MOESM3_ESM.pdf]

File name: Supplementary Data 1-2

Description:

Supplementary Data 1. Anhydrous major-element compositions of calculated melts.
Supplementary Data 2. Trace-element compositions of calculated melts.
